# Getting Serious about Shared Features

**DOI:** 10.1093/bjps/axy029

**Published:** 2018-06-28

**Authors:** Donal Khosrowi

## Abstract

In *Simulation and Similarity*, Michael Weisberg offers a similarity-based account of the model–world relation, which is the relation in virtue of which successful models are successful. Weisberg’s main idea is that models are similar to targets in virtue of sharing features. An important concern about Weisberg’s account is that it remains silent on what it means for models and targets to share features, and consequently on how feature-sharing contributes to models’ epistemic success. I consider three potential ways of concretizing the concept of shared features: as identical, quantitatively sufficiently close, and sufficiently similar features. I argue that each of these concretizations faces significant challenges, leaving unclear how Weisberg’s account substantially contributes to elucidating the relation in virtue of which successful models are successful. Against this background, I outline a pluralistic revision and argue that this revision may not only help Weisberg's account evade several of the problems that I raise, but also offers a novel perspective on the model–world relation more generally.

1 Introduction
2 Weisberg’s Feature-Sharing Account
3 What Is a Shared Feature?
  3.1 Identity
  3.2 Sufficient closeness
  3.3 Sufficient similarity
4 Turning Weisberg’s Account ‘Upside Down’
5 Conclusion

## 1 Introduction

Various authors in the philosophy of science literature have offered accounts of the model–world relation, which is the relation in virtue of which scientific models exhibit their predictive and explanatory capabilities. Similarity-based accounts assert that models have such capabilities in virtue of being sufficiently similar to their targets in certain relevant respects (Cartwright [1983]; Giere [1988], [2004], [2010]; Godfrey-Smith [2006]). In contrast to isomorphism-based accounts (Bueno *et al.* [2002]; da Costa and French [2003]), they purportedly capture several important intuitions: that the model–world relation is instantiated in degrees (Weisberg [2013], p. 154); that only relevant instances of the relation matter for successful representation (Weisberg [2013], p. 135); and that contextual factors such as modellers’ epistemic aims play important roles in scientific representation (Giere [2004], [2010]; van Fraassen [2008]; Mäki [2009]).

Despite these achievements, similarity-based accounts also face several criticisms. One is that they remain uninformative about how models’ similarity to targets facilitates their epistemic capabilities unless it is specified in virtue of what properties models are purportedly similar to their targets (Stefanov [2012], p. 72). Another pertinent criticism is offered by Paul Teller ([2001]), who argues that attempts to offer a general account of model–world similarity are misguided, as only the particular context of specific modelling activities can tell us what it means for models to be relevantly and sufficiently similar to their targets.

Weisberg ([2013]) offers an analysis of model–world similarity that aims to resist these concerns. On this account, model–world similarity is determined by the proportion of features that models share and do not share with their targets. According to Weisberg, his account not only accommodates several important intuitions, but is also superior to earlier accounts specifically in virtue of offering an explicit analysis of what model–world similarity supervenes on ([2013], pp. 143, 155).

Weisberg’s account has been criticized on several grounds. Tal ([2015]) worries that it involves a circularity. O’Connor and Weatherall ([2016]) argue that a single relation like similarity is unable to capture the substantial heterogeneity in how models across the sciences relate to their targets. Parker ([2015]) raises several, more specific concerns, one being that on Weisberg’s account it is unclear what form shared features take.

In what follows, I offer a line of criticism that significantly expands on Parker’s concern. Starting from the diagnosis that a fundamental shortcoming of Weisberg’s account is that it remains vague about what it means for models and targets to share features, I consider three ways shared features might be explicated: as identical, quantitatively sufficiently close, and sufficiently similar features. I argue that all three construals face significant challenges, leaving unclear how Weisberg can provide an informative account of the relation in virtue of which successful models are successful.

I then propose pluralistic revisions to Weisberg's account. This revised account offers a menu of different ways models can share features with their targets: by means of quantitative closeness, identity, isomorphism, similarity, and possibly other relations too. This revision does not only help Weisberg's account with some of the challenges that I raise, but also offers a novel perspective on the model–world relation more generally. Specifically, it departs from the monistic character of extant similarity- and isomorphism-based accounts that have specified one single type of relation to govern the manifold ways successful models can relate to their targets. The pluralistic revision I sketch out adds a conciliatory perspective, suggesting that there can be a division of labour between these rival accounts, where certain types of relations, such as isomorphism, similarity, identity, and quantitative closeness, are less or more appropriate for facilitating epistemic success depending on general characteristics of the modelling context, such as the type of model used, the kinds of features at issue, and the types of modelling goals being pursued. I argue that this pluralistic perspective offers a promising basis for improving our understanding of the relation in virtue of which successful models are successful.

Let me begin with a sketch of Weisberg’s account before I proceed to my critical and constructive contributions.

## 2 Weisberg’s Feature-Sharing Account

On Weisberg’s account, model–world similarity is a triadic relation between a model, a modeller, and a target system. According to Weisberg ([2013], pp. 39, 147), when constructing models, modellers pursue certain modelling goals regarding the model’s scope and fidelity desiderata such as predictive accuracy, robustness, explanatory power, among others. These goals inform in which respects models and targets are intended to be similar, and in which respects it is of no concern whether they are.

To capture this, Weisberg ([2013], p. 148) introduces the feature set, which contains the features of a model and its target that a modeller considers relevant for her modelling. The members of this set are divided into two types of features: attributes and mechanisms. Attributes are the features of a model or target that can be considered its states, and mechanisms are the transition rules that bring about these states ([2013], p. 145).

With this background in place, Weisberg’s main idea is that features that are shared by the model and target should add to their similarity, while features that are not shared should detract from it. This draws on an extensive tradition of explicating similarity in terms of shared properties (Hesse [1966], p. 71, [1974], p. 67; Goodman [1972]).

Building on this idea, Weisberg ([2013], p. 146) distinguishes two ways models and targets may fail to share features: first, features that a model exhibits but the target does not should detract from their similarity—call them extraneous features; second, features of a target that are not captured by the model should also detract from their similarity—call them missed features. Against this background, and drawing explicitly on an account by Tversky ([1977]), Weisberg ([2013], p. 147) proposes the following metric of model–world similarity that involves the purportedly clearer concepts shared, missed, and extraneous features:

$$S(M,T)=\frac{\left|M_{a}{\cap^{\text{​}}}^{\text{​}}T_{a}|+|M_{m}{\cap^{\text{​}}}^{\text{​}}T_{m}\right|}{\left|M_{a}{\cap^{\text{​}}}^{\text{​}}T_{a}|+|M_{m}{\cap^{\text{​}}}^{\text{​}}T_{m}|+|M_{a}-T_{a}|+|M_{m}-T_{m}|+|T_{a}-M_{a}|+|T_{m}-M_{m}\right|}.$$

Here, *M* refers to the model’s and *T* to the target’s features and the subscripts *a* and *m* partition features into attributes and mechanisms. In the numerator, we find the intersection of attributes and the intersection of mechanisms, which add to the cardinality of shared features. We find the same terms in the denominator. In addition, we find ${|M}_{a}-T_{a}|+{|M}_{m}-T_{m}|$, which count the model’s extraneous features, as well as $\left|T_{a}-M_{a}|+{|T}_{m}-M_{m}\right|$, which count the model’s missed features. An increase in either term yields a decrease in $S\left(M,T\right),$ penalizing features that are not shared. In the case of perfect similarity, they are empty sets, yielding a similarity value of unity. In the converse case of minimal similarity, the cardinality of features that are not shared will exceed the cardinality of shared features significantly. Hence, in the positive limit of these terms $S\left(M,T\right)$ approaches zero, so $S\left(M,T\right)\in \left[0,1\right]$ (Weisberg [2013], p. 147).

In addition to this calculus, Weisberg’s account also permits the use of term weights and weighting functions. Weighting functions capture the idea that modellers may consider some features to be more important than others, and term weights capture that modellers may wish to put more emphasis on some of the terms, such as in how-possibly modelling where shared attributes are emphasized over shared mechanisms (Weisberg [2013], pp. 151–2).

With this sketch in place, let me expand on Weisberg’s pursuit of at least three distinct aims. His first aim is to inform about the goodness of fit between models and targets. According to Weisberg ([2013], p. 150), his account can help scientists compare ‘the relationship that [a] model actually holds to the world to the one that they are interested in achieving’. Weisberg’s second aim is to inform about the sources of disagreement between scientists about the goodness of fit of models to targets. According to Weisberg, such disagreements can be explained as a function of differences in scientists’ feature sets, term weights, and weighting functions. The third, analytical aim is to offer a reductive analysis of what model–world similarity supervenes on (Weisberg [2013], pp. 143, 149, 155). This is accomplished by specifying several distinct realizers of similarity: instances of shared, missed, and extraneous features, as well as contextual and interest-relative factors, including modellers’ feature sets, term weights, and weighting functions.

In pursuing this analytical aim, Weisberg ([2015], p. 303) seeks to address the pertinent criticism that similarity is essentially vague, elusive, and may resist general philosophical analysis in principle (see, for example, Quine [1969]; Goodman [1972]; Teller [2001]). Applied to model–world similarity, this criticism amounts to arguing that as long as it is not explicated what model–world similarity consists in, claiming that successful models are successful in virtue of being highly similar to their targets does not elucidate the model–world relation beyond the level of intuitively appealing slogans. Weisberg ([2013], p. 143) seeks to evade this concern by offering an analysis of similarity in terms of the purportedly clearer concepts of shared, missed, and extraneous features.

Even so, while Weisberg explicitly presents his account as providing an analysis of similarity, it is unclear what the target of this analysis is. As Parker ([2015], p. 270) argues, Weisberg seems to equivocate between three distinct questions that such an analysis may seek to elucidate:

1. What is the relation in virtue of which successful models are successful?
2. What is the sort of relation that generally holds between models and targets?
3. What underlies scientists’ judgements of the extent to which models are similar to their targets?

In a reply, Weisberg ([2015], p. 300) clarifies that his account is ‘addressing all three of these themes’. In what follows, I focus attention specifically on the first aim: to provide an account of the relation in virtue of which successful models are successful. I take this to be the most salient and important issue that an account of the model–world relation should address. Moreover, I also consider this to be the intended target of earlier similarity-based accounts as well as other accounts of the model–world relation. Finally, Weisberg ([2013], p. 155) considers his account to be a substantive advancement over its intellectual progenitors specifically in virtue of offering an analysis that can better elucidate this question. With this in mind, let me turn to my main concern, which is that Weisberg remains vague about what shared features are.

## 3 What Is a Shared Feature?

While Weisberg is explicit concerning the distinction between missed and extraneous features, he does not explicate what shared features are and how instances of shared features can be distinguished from missed and extraneous cases.

There are several important questions here, including: (1) What is the distinction between shared and missed and extraneous features in general? (2) How do scientists determine whether a given feature is shared or not? (3) Is feature-sharing a gradual or dichotomous concept? (4) What is the role of background theory and modelling goals in determining whether features count as shared?

Without an explication of shared features that can address at least some of these questions, Weisberg’s account remains uninformative.^1^ It offers an analysis of similarity in terms of a purportedly clearer concept, feature-sharing, but this concept is no more clearly understood than similarity itself. So without an account of what shared features are, it remains unclear how and when they contribute to or account for models’ epistemic success, and Weisberg’s analysis subsequently fails to elucidate the relation in virtue of which successful models are successful.

This concern is further aggravated when considering Teller’s contextual view on model–world similarity. Teller ([2001], p. 402) argues that there can be no general analysis of model–world similarity as only the specific details of particular modelling contexts can tell us what it means for models to be relevantly and sufficiently similar to their targets. Applied to the issue of what counts as a shared feature, a Tellerian view might say that there can be no general analysis of feature-sharing as only the details of specific modelling contexts can tell us which features need to be shared, as well as in what ways and to what degrees they need to be shared.

On this view, there is nothing both general and informative to say about what feature-sharing consists in that could justify a general account of the model–world relation in terms of feature-sharing. So unless a way can be found to demonstrate that there are elucidatory means by which to structure and systematize the manner in which context matters for determining what counts as a shared feature, at some level of generality, this would seem to concede that it is the concrete context of particular modelling activities, rather than Weisberg’s general account, that elucidates the relation in virtue of which successful models are successful.

This seems undesirable. While the Tellerian view seems to get many things right—for instance, by highlighting that modelling practices across and within the sciences are sufficiently heterogeneous to suspect that providing a comprehensive account of some single relation in virtue of which successful models are successful is difficult—it is also a highly unsatisfactory view. Of course, the details of specific modelling contexts are important for clarifying in virtue of what relations to targets successful models are successful. Yet this does not imply that nothing general can be said about these relations. The challenge that this presents for Weisberg’s account then is to demonstrate that feature-sharing can be concretized in a way that is not fully context-specific and that can offer elucidatory insights about the model–world relation that neither the Tellerian appeal to context nor earlier, slogan-versions of similarity-based accounts can offer.

With this background in place, let me turn to examine three potential construals to concretize shared features: as identical, sufficiently close, and sufficiently similar features. I argue that each of these concretizations is individually insufficient for providing an informative and general account of the relation in virtue of which successful models are successful.

### 3.1 Identity

The first construal of feature-sharing says that shared features are those that are identical between a model and its target. This yields at least two undesirable consequences. The first is that it creates implausibly strong requirements for successful representational relationships. For instance, the identity construal suggests that a model’s variables need to assume all the same values as their referents in the target in order to share features with the target. On this construal, many models would incur significant penalties on their similarity to targets in virtue of the various idealizations and approximations that they employ. While these models may still be epistemically successful relative to a wide range of plausible modelling goals, they will typically fail to be identical to their targets in any respects, be that estimated parameters versus true underlying values of parameters or predicted values versus observed values (Suárez [2003], pp. 234–5). This suggests that many successful models would exhibit surprisingly low similarity values even though they may intuitively appear relevantly and highly similar to their targets. This is undesirable, as it would undermine Weisberg’s envisioned connection between similarity and epistemic success.^2^

In the same vein, the identity construal also renders Weisberg’s account vulnerable to some of the concerns that he offers against isomorphism-based accounts. Weisberg ([2013], p. 141) worries that these accounts impose exceedingly stringent requirements on representational relationships and fail several important desiderata for a persuasive account of such relationships. One of his main concerns is that many successful models fail to be isomorphic to their targets in any important respects ([2013], p. 140). Hence, these models would not be considered successful representations, even though they may issue accurate predictions, adequate explanations or exhibit other kinds of epistemic utility. Irrespective of whether one agrees with this criticism, Weisberg’s concern about exceedingly stringent constraints imposed by isomorphism-based accounts would seem to extend to his own account under the identity construal because, in many cases, exact identity between features seems to imply similarly stringent constraints on feature-level relations, precluding potentially many epistemically important feature-level relations from counting as shared features.

A related concern is offered by Parker ([2015]), who suggests that thinking of shared features as identical features prompts us to construe features in odd ways. Consider the case where a model’s parameter α is slightly inaccurately parameterized; say it is off by 0.1 units due to measurement error. Parker’s worry is that the identity construal would have us say that the model and target share a feature that is defined as ‘exhibiting a value of α that is within 0.1 of the true value of α in the target’. Parker ([2015], p. 273) argues that at least in some cases it seems more natural to understand shared features as sufficiently similar features. In a reply to Parker, Weisberg ([2015], p. 303) clarifies that his conception of feature-sharing allows us to think of shared quantitative features not as strictly speaking identical features (in the sense of full-on quantitative identity), but rather as features that are within some threshold range of each other, thus allowing quantitative features to count as shared even when they are not exactly identical. Let me expand on two additional construals of shared features in turn, which, I believe, capture Weisberg’s concretization.

### 3.2 Sufficient closeness

The sufficient closeness construal says that shared features are those that are sufficiently quantitatively close to each other on some subvenient scale. In other words, models’ features must be within some threshold range of corresponding features of the target to count as shared. While this seems to avoid the implausibly stringent demands of the identity construal, it creates two general problems.

The first problem is that it constrains the scope of Weisberg’s account to quantitative features: those that are operationalizable in terms of some quantitative scale that permits evaluation of absolute differences between features. Clearly, not all features are readily amenable to such operationalization. For instance, it is difficult to make sense of the idea that qualitative or categorical features need to be quantitatively sufficiently close to each other. Categorical features such as economic agents exhibiting rational preferences, or having quasilinear utility functions are not amenable to treatment in terms of sufficient quantitative closeness. Another important case is that of mechanisms, where it seems unclear how to determine whether a model’s mechanism is sufficiently quantitatively close to the mechanism that governs the phenomena of interest in the target.

The second, more general problem with the sufficient closeness construal is that it raises the question how the ranges are determined that distinguish between sufficient and insufficient closeness, and hence between shared and missed features. This is important as without providing more details on how these ranges are determined, Weisberg’s account would seem unable to tell us whether a given model and its target share features, and hence whether they are similar at all and, if so, to what extent.

A first pass at concretizing the sufficient closeness construal in this respect could be to say that modelling goals and background theory, including less fully articulated beliefs about the target, jointly inform or determine these ranges. So modelling goals would tell us what counts as epistemic success and background theory would help us determine whether certain ranges of closeness will be sufficient for promoting such success. For instance, physical background theory might give us reasons to believe that a scale model of an airplane in a wind tunnel will be useful for successfully predicting airplanes’ stall angle of attack only to the extent that the model’s (scaled) geometrical features are within some tightly defined range of closeness compared to corresponding features of the target plane. So at least in some cases, our understanding of the target is sufficiently sophisticated to give us a clear idea of just how close features need to be in order for epistemic success to ensue.

Even so, there are several reasons to be sceptical about whether this way of fleshing out the sufficient closeness construal will yield the account that Weisberg aims to offer. First, saying that context-specific factors such as modelling goals and our particular background theory of the target will help distinguish sufficiently and insufficiently close features would seem to concede to the Tellerian view that it is the concrete context of specific modelling activities that will tell us what degree of closeness between features will distinguish between shared and missed instances. This raises the question why we need a general account like Weisberg’s if it is context-specific information that elucidates the relation in virtue of which particular successful models are successful.

Moreover, context-specific information sufficient to distinguish shared and missed features is not always available, so even if Weisberg were to say that the modelling context, specifically our background theory of the target, will supply the requisite details for distinguishing shared and missed features, this would not help us much if there is no such background theory.

To illustrate, agent-based financial market models (see, for instance, Le Baron [2006]) are used to predict and explain phenomena such as asset pricing or stock market dynamics. The agents that figure in these models are characterized by features such as utility functions, updating rules and forecasting strategies. Such models consequently exhibit various parameters that need to be specified, such as agents’ risk preferences, learning speeds or their propensity to adopt other agents’ strategies. These parameters are usually calibrated by trying to replicate certain stylized features of the target.

But just how finely do the models’ parameters need to be tuned, and just how accurately must the model replicate known features of the target in order to be able to successfully predict or explain in other instances? This seems unclear as there is no background theory that tells us, for instance, that if we do not adequately calibrate model agents’ learning speed to within a certain threshold, then the model will be unable to issue accurate predictions of stock market behaviours. This is not surprising either, as it seems that in cases like this, where the target is not well understood, the understanding of the target required to determine thresholds for distinguishing shared and missed features amounts, in part, to the very understanding that the model is intended to facilitate in the first place.

In such cases, the sufficient closeness construal would seem to say little more about how relations of quantitative closeness promote models’ epistemic success beyond offering the generic suggestion that they need to be closer rather than less close, and preferably quite close, although we generally do not know just how close before the model’s epistemic capabilities are compromised. So unlike in the model airplane case, modelling goals and background theory do not always offer guidance for setting thresholds of sufficient closeness, and it remains unclear how shared and missed features can be distinguished.

Weisberg might reply that despite these challenges, there is nevertheless some fact of the matter as to which ranges of closeness are sufficient to promote models’ epistemic success and that a shared feature is one that conforms to this fact, even if we are not in the epistemic position to tell what the ranges are. However, this reply faces several problems of its own.

First, this would seem to beg the question whether feature-sharing is indeed the relation in virtue of which successful models are successful. While saying that shared features are all and only those that contribute to epistemic success would correctly single out those feature-level relations that promote our modelling goals, this trivializes the relation between feature-sharing and epistemic success because it fails to tell us whether a given feature is shared independently of whether it successfully promotes our goals. It is clear that the relation between feature-sharing and epistemic success needs to be specified, at least in part, according to criteria that do not presuppose the substantive relation between feature-sharing and epistemic success that Weisberg aims to establish.

Second, when laying out the desiderata for his account, Weisberg ([2013], p. 137) emphasizes that the similarity metric should be tractable in the sense that it ‘should reflect judgments that scientists can actually make, as opposed to asserting that the relation holds between inaccessible, hidden features of models and targets’. This suggests that Weisberg aims for an account that enables scientists (and perhaps philosophers) to tell whether a given model is similar to its target and to what extent. So by Weisberg’s own ambitions, it does not seem enough to suggest that there is some epistemically inaccessible fact as to whether a given feature is shared or not, and that reference to such fact gives us the desired account of the relation in virtue of which successful models are successful.

Third, at least in some cases, the very idea that there is some fact as to whether a specific range of closeness singles out features that promote epistemic success seems elusive. Consider models with multiple parameters such as the agent-based models outlined earlier. For one specific parameter, such as agents’ learning speeds, the question of whether certain values are sufficient to promote success will also hinge on the values of other parameters, such as agents’ risk preferences. If one of the model’s parameters is grossly mis-specified, such as when agents have implausible risk preferences, even exact identity in other parameters such as learning speed may not be sufficient to help modellers achieve any modelling goal. This is despite the model sharing many features, and thus exhibiting high degrees of similarity. This suggests that the way in which ranges of sufficient closeness are determined may also need to accommodate such interdependencies.^3^ But this makes matters even more challenging, since we would need to know a great deal about the target in order to tell which combinations of parameter values are jointly sufficient to render a model epistemically successful.

I take these concerns to indicate that the sufficient closeness construal is not adequately concretized by pointing out that some conjunction of background theory and modelling goals will take care of telling us when features are sufficiently close. So without further concretization, the sufficient closeness construal, by itself, seems insufficient to accomplish Weisberg’s aim of providing an informative account of the relation in virtue of which successful models are successful. Let me focus attention on a third construal of shared features.

### 3.3 Sufficient similarity

The third construal says that shared features are those that are sufficiently similar to each other. For instance, an agent-based financial market model that can approximately replicate a certain dynamical feature of the target (for example, a time-series characteristic such as leptokurtic returns distributions) may be considered sufficiently similar to the target with respect to that feature, and hence share the feature, whereas if it fails to be similar, this will yield a missed feature. This way of construing shared features again creates problems.

If Weisberg accepts the construal, then his analysis remains uninformative because it tells us that what it means for models to be similar to their targets is that they are similar in certain important respects, not too dissimilar in too many other important respects, while they may be similar or dissimilar in any number of unimportant respects. This does not elucidate the relation in virtue of which successful models are successful.^4^ If Weisberg sets out to analyse model–world similarity in terms of feature-sharing, and shared features are explicated as sufficiently similar features, then this just prompts all the same concerns that have affected earlier accounts of model–world similarity. While Weisberg’s account may still be taken to offer interesting ideas on how the global similarity of models to targets hinges on local, feature-level similarities, as well as interest-relative factors such as feature-weights, the feature-level similarities would remain a black box, and there do not seem to be any accounts of feature-level similarity that offer a remedy for this.

This does not mean, however, that the sufficient similarity construal is implausible. It seems that at least for some kinds of features and some kinds of modelling activities, shared features are best understood as sufficiently similar features.

The most general case is that of qualitative features; features that are vaguely defined and either do not permit of quantitative operationalization at all (at least not in useful or cognitively tractable ways) or are not operationalized in this way. Examples for this type of feature are descriptions of shapes and structures, such as saying that DNA strands are connected by a *T*-shaped juncture, complex dynamical features and patterns, as well as categorical features such as morphological or behavioural characteristics that are multiply realizable.

If there is no useful way to quantitatively operationalize qualitative concepts such as *T*-shapedness of DNA junctions, but such features are considered epistemically relevant, then it seems that the question of whether model and target share the feature should be understood either as a question of whether they are identical or whether they are similar in this respect.

I take it that construing shared qualitative features as identical features will often be difficult because qualitative concepts are usually vaguely defined. If features such as *T*-shapedness are vaguely defined, then at least some instances of *T*-shapedness on the part of a model may be epistemically uninteresting but still count as identical and hence shared. This would undermine the substantive connection between feature-sharing and epistemic success that Weisberg aims to establish. Likewise, if *T*-shapedness is more narrowly defined, then this might exclude epistemically beneficial instances from the realm of shared features because the requisite features on the model’s part will fail the definition. This would also undermine the connection between feature-sharing and epistemic success, because successful models would exhibit fewer feature-sharing relations. This suggests that it is difficult to handle qualitative features under the identity construal, since feature semantics would have to be constantly adapted in order to match our judgments of whether identity in these features, so defined, promotes epistemic success.

This problem can be sidestepped by saying that the model and target are sufficiently similar with respect to features such as *T*-shapedness. This allows answering questions about how similar model and target are, and whether such similarity is sufficient for attaining our modelling goals in two separate steps. I take this to be a strong reason in favour of construing shared qualitative features as sufficiently similar features.

The sufficient similarity construal may also be a useful way to express what it means that models and targets share mechanisms. Neither the identity nor the sufficient closeness construal seem apt to capture the kinds of relations that need to obtain between models’ and targets’ mechanisms in order for models to represent their targets successfully. For instance, for many epistemic purposes in social and behavioural sciences it is sufficient to model mechanisms at relatively high levels of abstraction intended to capture key qualitative causal relationships. This proceeds without making attempts to ensure that all causal relationships relevant to the phenomena of interest, but not necessarily relevant for the particular epistemic activity at issue, are encoded in the model’s mechanism. In such cases, mechanisms may be considered sufficiently similar at some level of abstraction relevant to the modeller’s epistemic interests, where sufficient similarity may be further explicated in terms of partial identity or partial similarity in components of the mechanisms at issue.

Even so, while at least in some cases the sufficient similarity construal seems to be a plausible way to construe feature-sharing relations (for instance, when qualitative features or mechanisms are at issue, targets are not well understood and background theory and sophisticated understanding of the target are unavailable), this construal also faces essentially the same challenges that affect the sufficient closeness construal. Saying that (some kinds of) shared features are sufficiently similar features can only yield an informative analysis of the relation in virtue of which successful models are successful if it says at least something about the distinction between sufficiently and insufficiently similar features, and preferably also something on how scientists can determine whether features are sufficiently similar.

As anticipated, there are two important challenges here. First, spelling out whether features are sufficiently similar should not beg the question whether sufficiently similar features contribute to epistemic success. So it is not enough to say that features are sufficiently similar whenever they contribute to epistemic success and insufficiently similar otherwise. Second, the sufficient similarity construal would also need to specify what sufficient similarity amounts to, at least partly, in general rather than context-specific terms. This is to push back on the Tellerian view that it is context-specific information rather than a general account like Weisberg’s that tells us what it means for models to be sufficiently similar on the level of particular features.

In summary, the above concerns suggest that, at least in its present form, Weisberg’s account does not provide an informative analysis of the relation in virtue of which successful models are successful that critics of similarity-based accounts have called for and that Weisberg promises to offer. Such an account should not bottom out at the level of a feature-sharing metric that does not concretize what shared features consist in, but should provide more details about the feature-level relations that can constitute instances of shared features. As I have argued, however, doing so raises at least three significant challenges.

The first is about scope: some kinds of feature-sharing relations seem best understood in terms of similarity, whereas others might be better understood in terms of sufficient closeness or identity. Each of these relations seems appropriate for some types of cases, but none of them seems privileged in the sense of accounting for a majority of feature-sharing instances. The second challenge, posed by the Tellerian view, is that pointing to contextual factors to help distinguish shared and missed features, without further general analysis, would boil down to saying that contextual information is what elucidates the relation in virtue of which successful models are successful. This leaves unclear why we need a general account like Weisberg’s for this purpose. The third challenge is concerned with establishing Weisberg’s envisioned connection between feature-sharing and epistemic success in a way that does not beg the question whether shared features, in any of the senses outlined above or others, contribute to epistemic success. Together with the second challenge, this creates a tension between saying too little about when and how certain feature-level relations contribute to epistemic success and, on the other hand, relegating this important issue to be settled by the contextual details of each particular modelling activity, thus leaving it unclear how Weisberg’s account substantially contributes to elucidating the relation in virtue of which successful models are successful.

I consider this situation unsatisfactory, which is why I now want to offer some constructive suggestions for how Weisberg's account might be revised, adopting a pluralistic account of feature-sharing instead. I argue that doing so can evade at least the first two of the above challenges, and may partly address the third. While I do not aim to fully develop a pluralistic revision of Weisberg’s account here, I hope that my sketch of this revision will make clear that it offers a promising option the account, as well as a novel perspective on the model–world relation more generally.

## 4 Turning Weisberg’s Account ‘Upside Down’

As the preceding discussion indicates, different kinds of feature-level relations seem suitable for elucidating the relation in virtue of which successful models are successful depending on the kinds of features, models, and modelling goals at issue. This suggests that a version of Weisberg’s account that further concretizes the idea of feature-sharing should be pluralistic about the kinds of relations that can instantiate shared features. To accommodate this pluralism, my key suggestion is to turn Weisberg’s account ‘upside down’ and say that the relation in virtue of which successful models are successful, at the most general level, is one of feature-sharing instead of similarity. So rather than starting from the slogan that successful models are successful because they are highly similar to targets, and providing a reductive analysis of similarity in terms of feature-sharing, the idea is to begin from (weighted) feature-sharing as the general relation in virtue of which successful models are successful and take a pluralistic view concerning how shared features can be instantiated: as identities, similarities, sufficient quantitative closeness, isomorphisms, and possibly other relations.

With this menu of relations in place, a pluralistic version of Weisberg’s account helps concretize how the suitability of different kinds of feature-level relations changes with respect to the kinds of features, models, and modelling goals at issue, as well as our understanding of the model and the target. As I have argued in Section 3, each of the construals considered there seem useful for some kinds of models, features, and modelling goals, but not others. A pluralistic account builds on this by allowing that one and the same model can relate to its target in several different ways at once. For instance, a model may exhibit (partial) identities in some respects (such as mechanisms) and sufficient quantitative closeness or similarities in others (such as parameters and categorical characteristics). This seems useful, as different feature-types often seem to require different kinds of relations to express how models successfully relate to their targets on the level of such features. Qualitative features seem best understood in terms of similarity, while quantitative ones seem better understood in terms of sufficient closeness. Categorical features might be understood in terms of identity, and more complex structural features might be best captured by isomorphisms. This is how differences in feature-kinds induce differences in how feature-sharing is best characterized.

A pluralistic account is not only responsive to the nature of the features at issue, but also to the modelling goals underlying the modelling activity in question. It permits that one and the same feature pair can require different kinds of relations depending on general contextual factors, such as the kinds of modelling goals and the envisioned fidelity of the kinds of inferences that the type of model is intended to facilitate. This, too, seems useful, as it is plausible to think that the types of feature-level relations that are suitable to promote epistemic success, as well as the required fidelity of such relations, vary with respect to the kinds of modelling goals pursued.^5^ For instance, ambitious goals concerning predictive accuracy may require more stringent constraints on what counts as a shared feature, such as tight ranges of quantitative closeness or sufficient similarity in precisely characterized qualitative features.

A pluralistic feature-sharing account hence provides significantly more resources and flexibility to flesh out the details of how general characteristics of the modelling context constrain and determine the particular ways in which, and degrees to which, models need to share features with their targets in order to be epistemically successful.

How much does this move to pluralism depart from Weisberg’s initial project, then? Very little in some respects, more dramatically in others. For once, a move to pluralism still allows Weisberg's account to retain its core: the (weighted) feature-sharing metric. This seems useful as it already captures several important intuitions about the model–world relation: that it is gradual, that only relevant instances matter for epistemic success, and that contextual factors such as modelling goals and judgments about the relative importance of features play important roles in scientific representation.

At the same time, there are also significant departures involved in a pluralistic revision. Weisberg’s metric would not figure as a metric of similarity anymore, but only as a metric of the (weighted) degree of feature-sharing between models and targets, which is now the principal relation in virtue of which successful models are successful. Importantly, turning Weisberg’s account ‘upside down’ involves abandoning the aim of analysing away similarity as an uninformative and too general a gesture that, at best, summarizes but does not elucidate the manifold ways successful models relate to targets. As I have argued, this does not seem to be the right way to go. There are cases where it seems highly plausible to say that on the level of features, models can (or should) relate to their targets by means of similarity, such as when features are qualitative or vaguely defined, desired levels of inferential fidelity are modest, targets are not well understood, and strong background theory is unavailable. So contra Weisberg, as well as many critics of similarity, a pluralistic account maintains that similarity has a legitimate role to play in philosophical accounts of the model–world relation, not as the principal relation in virtue of which successful models are successful, but as one among several other relations that models exhibit vis-à-vis their targets on the level of features. So, similarity should figure as a legitimate component of an account that maps out several conceptually distinct kinds of feature-level relations that are differentially suitable depending on the general and context-specific features of different modelling activities.

As such, similarity will perhaps remain a conceptual primitive, in line with the concerns articulated by many of its critics. This does not mean, however, that similarity, on the feature level, will be devoid of explanatory power. For instance, saying that a DNA computer model used for qualitative prediction of DNA strand displacement kinematics needs to be sufficiently similar to its target with respect to features such as *T*-shapedness of specific DNA junctures can provide more detail about what it means to share the feature in question than appealing to an unanalysed notion of feature-sharing to account for models’ epistemic success. Specifically, saying that feature-level similarity is the appropriate feature-level relation for the type of case at hand can now proceed against a richer background where similarity is characterized as the appropriate relation for the epistemic purposes at issue specifically because *T*-shapedness is a vaguely defined, but otherwise well-understood, qualitative feature. This not only provides more detail about what kind of feature-level relation is relevant for epistemic success, but can also tell us why similarity, rather than some other relation, is the appropriate kind of relation for the type of case at hand. The general aspects of the modelling context, such as the nature of the features at issue (qualitative), and the types of modelling goals pursued (for instance qualitative prediction of DNA strand displacement kinematics) can help us appreciate why, in the type of case at hand, similarity in features is all we need for epistemic success to be likely, or, in other cases, perhaps all we may hope for. So at least in cases where this is so, feature-level similarity can play a legitimate role in an account of the model–world relation. This is how my suggestion to understand feature-sharing as a general relation that can be instantiated by several more specific relations, including similarity, departs importantly from Weisberg’s initial project.

Let me expand on how a move to pluralism helps address some of the challenges for Weisberg's account that I have discussed. The first, immediate advantage is that it helps alleviate concerns about scope: none of the construals of feature-sharing considered above seem individually sufficient to handle the full gamut of feature-types that models may share with their targets. A pluralistic account can address this by offering a menu of such construals and by elaborating how their suitability varies with respect to the kinds of features, models, and modelling goals at issue. The construals outlined here may serve as a starting point, but a concretization of Weisberg’s account is, of course, not limited to just those relations I have examined. Other relations, such as isomorphisms, suggest themselves as well. This is interesting because in contrast to earlier, monistic accounts of the model–world relation in terms of either similarity or isomorphism, a pluralistic revision carves out space for each of these relations as complementary, rather than rival accounts of how models relate to targets on the level of features.^6^ It thus offers a conciliatory perspective on these relations and an opportunity for their proponents to specify the conditions under which they consider these relations to be particularly suitable to capture how successful models relate to their targets.

The second way a move to pluralism helps evade the challenges raised above is that it helps push back on the Tellerian view. As I have argued, an account of feature-sharing should not boil down to saying that it is the specific context of particular modelling activities that will tell us what constitutes a shared feature. This would raise the question of why we should have an account like Weisberg’s at all if what eventually does the work the account is supposed to do is context-specific information. To be sure, this would not be a problem if the Tellerian view were a satisfactory position. But I do not think that it is. I consider the Tellerian view to be an unsatisfactory view as it suggests that there can be no general taxonomy of different types of models and modelling strategies that can successfully single out epistemically significant commonalities of tokens of these types with respect to how models relate to targets and in virtue of what kinds of relations they tend to be epistemically successful. It seems that there are ways, even at relatively coarse-grained levels of classification, to distinguish between different types of modelling activities concerning the respects in which, the particular ways in which, and the degrees to which models involved in these activities need to be suitably related to targets for epistemic success to be likely.

Weisberg’s account already covers important ground here. For instance, when elaborating the role of term weights, Weisberg illustrates how relatively general aspects of modelling activities help concretize the respects in which feature-sharing relations need to obtain. For instance, Weisberg ([2013], p. 151) argues that hyper-accurate modelling requires extensive feature-sharing in both attributes and mechanisms, whereas how-possibly modelling generally disregards shared mechanisms and focuses on the replication of known attributes of the target. In addition to term weights, the weighting functions included in Weisberg’s account further concretize the respects in which feature-sharing relations need to obtain. As Weisberg suggests, relatively general background theory can offer additional constraints on the kinds of features for which feature-sharing relations are particularly important. These constraints, too, can be formulated generally rather than applying only to particular modelling contexts. Such distinctions about what terms of the similarity metric, and what particular features, tend to be more important with respect to general differences in the kinds of modelling goals pursued push back on the Tellerian view already: they seem sufficient to grant that it is possible to say some interesting and general things about the respects in which models used in certain types of modelling activities need to share features with their targets.

At the same time, as I have argued at length, Weisberg’s account still falls short of elucidating the ways in which or degrees to which models need to be suitably related to targets in those relevant respects in order for epistemic success to be likely. This is because knowing what the respects are in which feature-sharing would be important for epistemic success does not, by itself, tell us what kinds of relations need to obtain between the features in question for them to count as shared and to promote epistemic success. A pluralistic account of feature-sharing can offer help here, as it provides more resources to specify what kinds of feature-sharing relations and what degrees of such relations (if applicable) seem suitable under varying conditions concerning the types of models, features, and modelling goals at issue.

This can proceed at different levels. For instance, mirroring Weisberg’s elaborations, hyper-accurate modelling may require higher fidelity of feature-level relations to promote epistemic success. This could mean that mechanisms need to be identical, rather than broadly similar, and that parameters need to exhibit high, rather than low degrees of quantitative closeness. How-possibly modelling may involve various different relations on the level of attributes depending on the specificity of the phenomena one is interested in replicating. For instance, for some Schelling-type agent-based models, rough similarities or broad ranges of closeness in attributes such as degree of segregation may suffice to serve modellers’ envisioned goals. In other cases, where explananda phenomena are more precisely characterized, feature-sharing may require more sophisticated relationships, such as sufficient closeness in several quantitative features at once.^7^

Such distinctions may proceed at finer-grained levels of classification as well, extending to model-types that are individuated not by the general purposes of the modelling activity in which they figure but by their type-specific characteristics. For instance, parametric structural equation models used in social sciences are constructed to represent causal mechanisms by specifying a set of variables and equations that characterize the functional relationships among these variables. These models are used for a variety of epistemic purposes, such as accurate prediction of the quantitative causal effects of counterfactual interventions. For such purposes, it often seems that nothing short of identity in all parts of the mechanism relevant to the production of the outcome variables of interest will be enough for the model to help achieve modellers’ intended goals. The model’s parameters, too, will need to be accurately specified for each of the equations that determine the relations between the model’s variables. This is in contrast to qualitative causal models that may generally address the same overall themes, albeit with significantly different intended levels of fidelity. Here, it is perhaps sufficient that models share the most important causal relationships that figure in the target’s mechanism, and that the signs of these relationships are correctly specified; but there is generally no concern for accurately estimating parameters or even more complex non-linear relationships. This suggest that qualitative similarity, rather than identity and maximal quantitative closeness, seem more suitable to capture the relations that are important for achieving the kinds of modelling goals for which these models are used.

It is important to recognize that these differences in what constitutes shared features are general in nature and apply to a class of models, rather than to individual models on a case-by-case basis. While all aspects that constrain or determine what counts as a shared feature will of course always be instantiated by some concrete context, this does not mean that they are context-specific. Rather, many of these aspects will generalize readily over different instances of models or modelling goals. It does not seem to matter, for instance, whether one is investigating the effect of class size on students’ performance, the clinical effectiveness of medical treatment on causally heterogeneous populations, or the effects of insecticide-treated bed nets on malaria infection rates. In each of these instances, the type of model used and the type of aim pursued by modellers constrain to a significant extent the appropriate ways models must relate to their targets on the level of features. What counts as a shared feature in each of these cases is not constrained by information that is idiosyncratic to any particular modelling context, as the Tellerian view would suggest, but by general aspects that are instantiated by each of the specific contexts.

Indeed, the context-specific aspects of modelling activities rarely seem to tell us many interesting things about what counts as a shared feature on their own. There is nothing about investigating the effects of class size on students’ performance that, by itself, tells us what kinds of feature-level relations will bear favourably on the epistemic success of a model chosen to investigate this effect. Instead of originating from context-specific aspects, such insights will typically be supplied by a conjunction of domain-general theory, our understanding of the kinds of models that we use, and general aspects of the modelling activity, such as the types of features and modelling goals involved. Of course, context-specific aspects can supply additional constraints on how models need to be related to their targets; think for instance about the context-specific epistemic risks involved in making decisions based on a model’s predictions. However, it seems that such details do not, by themselves, tell us what kinds of feature-level relations promote epistemic success. So context matters but, *pace* Teller, not only context matters for providing an account of how successful models relate to their targets, and contextual information alone does not always yield such an account on its own.

A pluralistic version of Weisberg’s account then allows us to take a conciliatory view on the tension between general accounts like Weisberg’s and the Tellerian view. ‘Conciliatory’ here means that there is a division of labour between them in that up to some point, relatively general aspects of modelling activities constrain, but perhaps do not fully determine, what counts as a shared feature, and context-specific details will allow us to fill in the remaining blanks if and when such information is available. How this labour will be divided, and whether a general and pluralistic version of Weisberg’s account can push back on the Tellerian appeal to context by a significant margin, is an open question that a more fully developed version of this proposal will need to investigate. Irrespective of the degree to which such an account succeeds in this respect, it may be elucidatory in itself to see just where the boundaries of general accounts and the concrete details of particular modelling contexts will meet, to jointly demonstrate the relation in virtue of which successful models are successful.

Finally, let me briefly comment on what a move to pluralism will do for addressing the third challenge, namely, that in concretizing the conditions under which features are sufficiently close or similar, there is the danger of begging the question on whether shared features contribute to epistemic success. As I have argued, clarifying what constitutes a shared feature must be settled independently of the eventual epistemic success that shared features are supposed to bestow on models. A pluralistic revision does not seem to offer much of a remedy. While it provides more resources to elaborate on the relative suitability of certain kinds of feature-level relations for certain kinds of models, features, and modelling goals, a good chunk of this problem may remain untouched, particularly in those recalcitrant cases where background theory and our understanding of the target are highly limited. Here, even a pluralistic account may need to bite the bullet and say that while there may, in principle, be more specific constraints on what counts as a shared feature, these constraints will not always be accessible to scientists and philosophers at satisfactorily precise levels.

In such cases, the context-specific details of a particular modelling activity, too, may be insufficient to shed more light on what shared features are. When background theory and understanding of the target are limited, it seems that, at least transiently, we may be unable to tell what constitutes a shared feature. So even a Tellerian view may need to concede that whenever the concrete details of the case will leave it unclear whether a feature is sufficiently similar or close before the eventual epistemic success or failure becomes apparent, an account of the relation in virtue of which these particular successful models are successful remains elusive.

This is perhaps not a surprising conclusion, however. In such cases, it seems that elucidating what sorts of feature-level relations promote epistemic success is among the reasons that scientists build models in the first place, namely, to develop a cumulatively more sophisticated understanding of what works and what does not by means of trial and error, rather than by means of antecedent guidance concerning what is already understood to render a model successful. In these cases, perhaps the best that philosophers interested in the model–world relation can hope for is that eventually, and after the fact, an account like Weisberg’s, together with the context-specific details of the particular case, will help us make sense of why epistemic success ensues, if and when it does. However, this, too, can be part of a revised version of Weisberg’s account, in that it may elaborate how our understanding of the target bears on our ability to distinguish shared and missed features at all.

This makes clear that a pluralistic revision of Weisberg’s account will not fully evade the challenges I have raised here. Despite this, it may still offer an interesting starting point for addressing some of them, as well as for thinking more generally about how these challenges, and the pluralistic character of the view I have sketched, bear on the prospects of providing more informative philosophical accounts of the model–world relation.

## 5 Conclusion

I have argued that Weisberg’s weighted feature-sharing account does not provide an informative analysis of similarity as the relation in virtue of which successful models are successful. I began by highlighting an important vagueness concerning what feature-sharing consists in. I then examined three construals of how this concept may be concretized and argued that they face several important challenges. This made clear that none of these off-the-shelf explications can individually render Weisberg’s analysis informative about the issues it is intended to elucidate.

Against this background, I sketched out a pluralistic revision of Weisberg’s account as a promising option to improve on this situation. By drawing on the joint resources of several more specific construals of feature-level relations, a pluralistic revision promises to offer more insights about how general aspects of modelling activities constrain and determine the ways features need to be shared. Moreover, such a revision provides a doubly conciliatory view: first, concerning the tension between general accounts like Weisberg’s and contextual accounts like the Tellerian view; and, second, concerning similarity and isomorphism-based contenders for a general account of the model–world relation. In virtue of this conciliatory character, I hope that this proposal helps stimulate future contributions aimed at improving our understanding of the relation in virtue of which successful models are successful.

## Footnotes

1 One might worry that it is important to keep the first two questions distinct, because an informative account may well be one that addresses (1) but not (2). I agree, but will not consider this here for three reasons: First, Weisberg’s account is not informative about either issue, so the distinction is immaterial to diagnosing its shortcomings. Second, my call for a more informative account is satisfied by a concretization of feature-sharing that addresses (1) but not (2). Third, Weisberg aims for an account that can address both (1) and (2). I thank an anonymous referee of this journal for emphasizing the need to clarify this.

2 One might think that given a suitable weighting function, even models with few remaining shared features can still be highly similar to targets. However, it is unclear whether models in general only need to share few truly important features. Moreover, the importance of features does not determine what the appropriate (or feasible) feature-level relations for these features are. So the remaining shared features are not necessarily important, nor are important features generally more likely to be shared, or best understood as shared, in the restrictive sense of identity. I thank an anonymous referee of this journal for raising this important point.

3 See (Parker [2015]) for a related concern about interdependencies between feature weights.

4 See (Tal [2015]) for a related concern about circularity.

5 This is in line with Parker’s ([2015], p. 275) suggestion for how Weisberg’s account could be concretized. It also seems to address O’Connor and Weatherall’s ([2016], p. 625) concern that a single relation like similarity is unlikely to accommodate the substantial heterogeneity in how successful models relate to their targets. Finally, my proposal is also compatible with the pluralistic spirit of Suárez’s ([2015]) deflationary account of representation. It would be incompatible, however, with a deflationary view on what constitutes successful representational relationships as I maintain that it is possible to spell out general constraints on what constitutes such relationships on the feature level.

6 While isomorphism-based accounts can be understood as a way of explicating what it means that models are similar to targets, at least some authors, including Weisberg, seem to think that isomorphism and similarity-based accounts are rival accounts of the model–world relation.

7 See (Bokulich [2014]) for a discussion of levels of abstraction in how-possibly modelling.

## References

[axy029-B1] Bokulich A. [2014]: ‘How the Tiger Bush Got Its Stripes: “How Possibly” vs. “How Actually” Model Explanations’, Monist, 97, pp. 321–38.

[axy029-B2] Bueno O., French S., Ladyman J. [2002]: ‘On Representing the Relationship between the Mathematical and the Empirical’, Philosophy of Science, 69, pp. 452–518.

[axy029-B3] Cartwright N. [1983]: How the Laws of Physics Lie, Oxford: Oxford University Press.

[axy029-B25] da Costa N., French S. [2003]: Science and Partial Truth: A Unitary Approach to Models and Scientific Reasoning, Oxford: Oxford University Press.

[axy029-B4] Giere R. N. [1988]: Explaining Science: A Cognitive Approach, Chicago, IL: University of Chicago Press.

[axy029-B5] Giere R. N. [2004]: ‘How Models Are Used to Represent Reality’, Philosophy of Science, 71, pp. 742–52.

[axy029-B6] Giere R. N. [2010]: ‘An Agent-Based Conception of Models and Scientific Representation’, Synthese, 172, pp. 269–81.

[axy029-B24] Godfrey-Smith P. [2006]: ‘The Strategy of Model-Based Science’, Biology and Philosophy, 21, pp. 725–40.

[axy029-B7] Goodman N. [1972]: ‘Seven Strictures on Similarity’, in his Problems and Projects, Indianapolis: Bobbs-Merril.

[axy029-B8] Hesse M. B. [1966]: Models and Analogies in Science, Notre Dame, IN: University of Notre Dame Press.

[axy029-B9] Hesse M. B. [1974]: The Structure of Scientific Inference, London: Macmillan.

[axy029-B10] Le Baron B. [2006]: ‘Agent-Based Financial Markets: Matching Stylized Facts with Style’, in Colander D. (*ed*), Post Walrasian Macroeconomics: Beyond the Dynamic Stochastic General Equilibrium Model, New York: Cambridge University Press, pp. 221–35.

[axy029-B11] Mäki U. [2009]: ‘MISSing the World: Models as Isolations and Credible Surrogate Systems’, Erkenntnis, 70, pp. 29–43.

[axy029-B12] O’Connor C., Weatherall J. O. [2016]: ‘Black Holes, Black-Scholes, and Prairie Voles: An Essay Review of *Simulation and Similarity*, by Michael Weisberg’, Philosophy of Science, 83, pp. 613–26.

[axy029-B13] Parker W. [2015]: ‘Getting (Even More) Serious about Similarity’, Biology and Philosophy, 30, pp. 267–76.

[axy029-B14] Quine W. V. O. [1969]: ‘Natural Kinds’, in his Ontological Relativity and Other Essays, New York: Columbia University Press.

[axy029-B15] Stefanov A. [2012]: ‘Theoretical Models as Representations’, Journal for General Philosophy of Science, 43, pp. 67–76.

[axy029-B16] Suárez M. [2003]: ‘Scientific Representation: Against Similarity and Isomorphism’, International Studies in the Philosophy of Science, 17, pp. 225–44.

[axy029-B17] Suárez M. [2015]: ‘Deflationary Representation, Inference, and Practice’, Studies in History and Philosophy of Science, 49, pp. 36–47.

[axy029-B18] Tal E. [2015]: ‘Review: Michael Weisberg, *Simulation and Similarity: Using Models to Understand the World*’, British Journal for the Philosophy of Science, 66, pp. 469–73.

[axy029-B19] Teller P. [2001]: ‘Twilight of the Perfect Model Model’, Erkenntnis, 55, pp. 393–415.

[axy029-B20] Tversky A. [1977]: ‘Features of Similarity’, Psychological Review, 84, pp. 327–52.

[axy029-B21] van Fraassen B. [2008]: Scientific Representation: Paradoxes of Perspective, Oxford: Oxford University Press.

[axy029-B22] Weisberg M. [2013]: Simulation and Similarity: Using Models to Understand the World, Oxford: Oxford University Press.

[axy029-B23] Weisberg M. [2015]: ‘Response to Critics’, Biology and Philosophy, 30, pp. 299–310.

